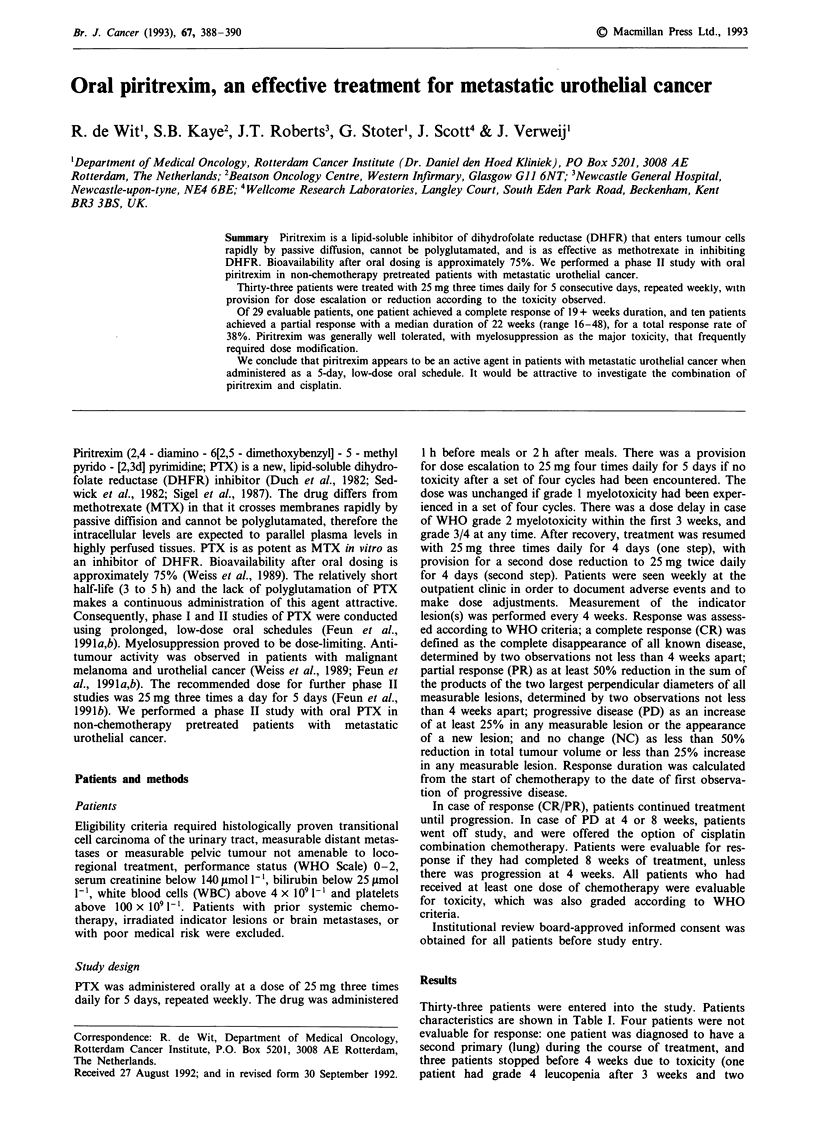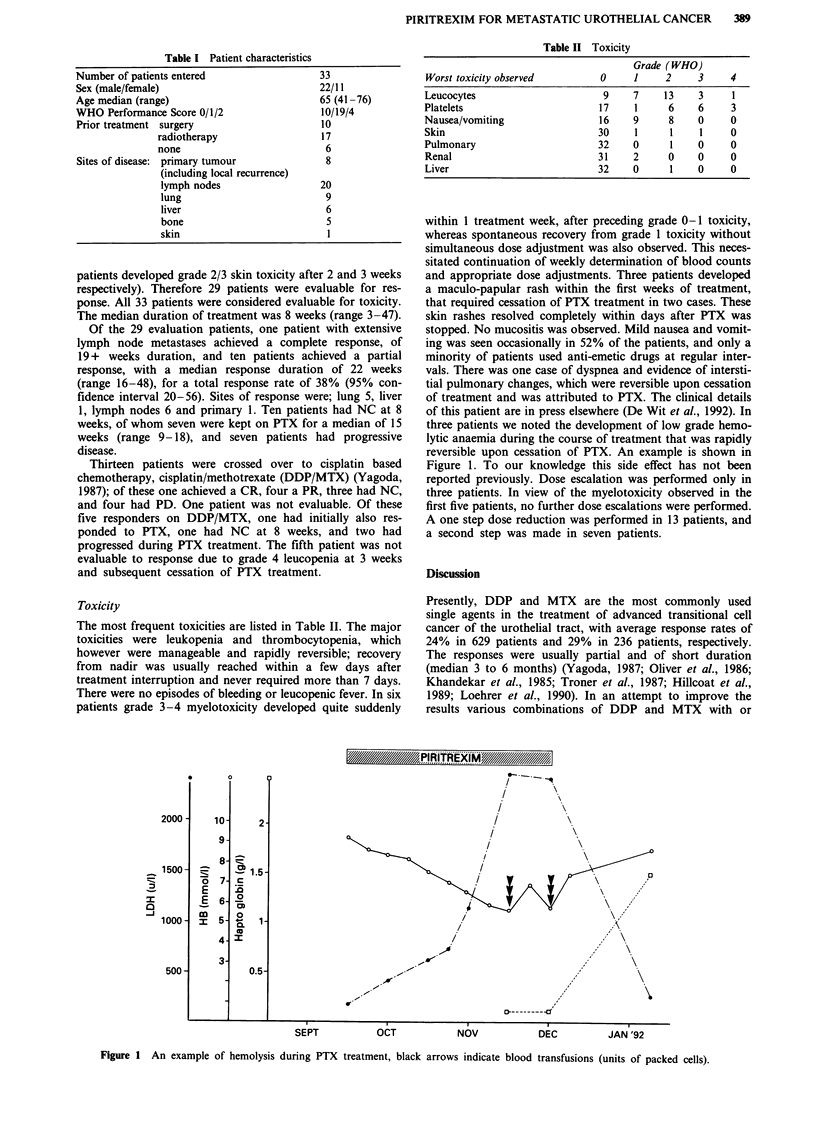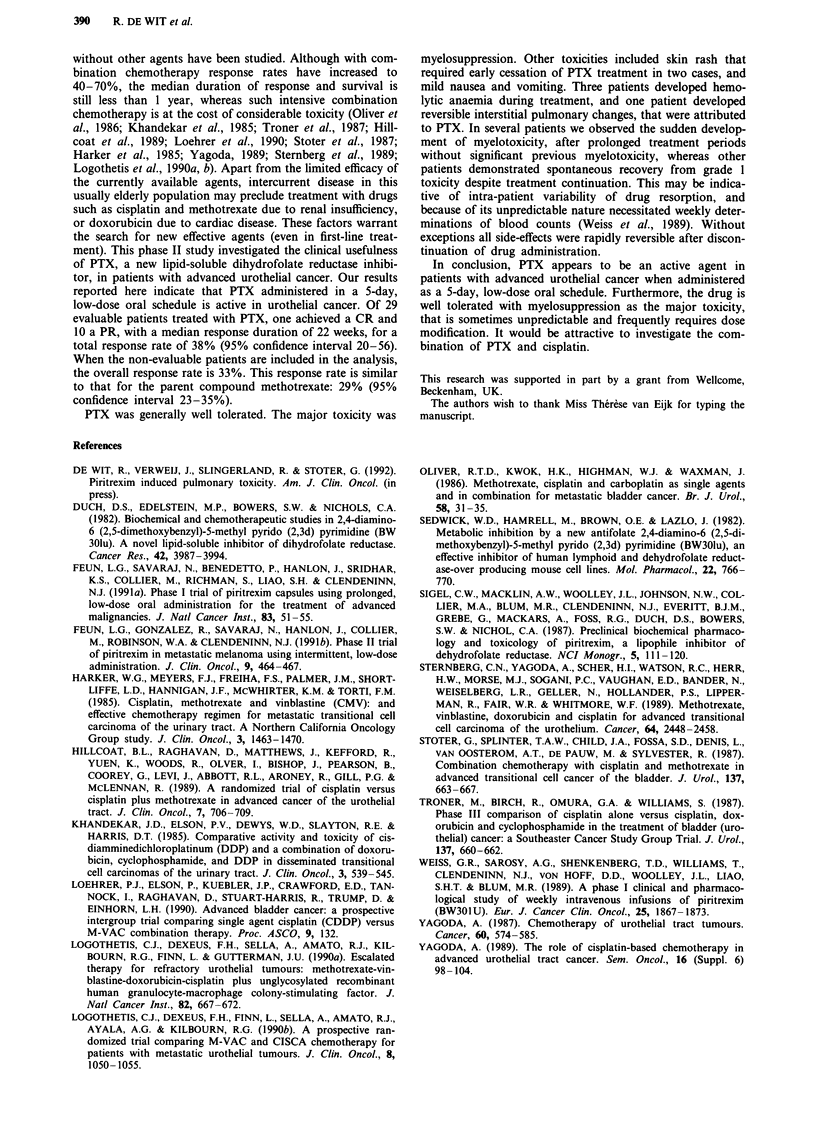# Oral piritrexim, an effective treatment for metastatic urothelial cancer.

**DOI:** 10.1038/bjc.1993.71

**Published:** 1993-02

**Authors:** R. de Wit, S. B. Kaye, J. T. Roberts, G. Stoter, J. Scott, J. Verweij

**Affiliations:** Department of Medical Oncology, Rotterdam Cancer Institute (Dr. Daniel den Hoed Kliniek), The Netherlands.

## Abstract

Piritrexim is a lipid-soluble inhibitor of dihydrofolate reductase (DHFR) that enters tumour cells rapidly by passive diffusion, cannot be polyglutamated, and is as effective as methotrexate in inhibiting DHFR. Bioavailability after oral dosing is approximately 75%. We performed a phase II study with oral piritrexim in non-chemotherapy pretreated patients with metastatic urothelial cancer. Thirty-three patients were treated with 25 mg three times daily for 5 consecutive days, repeated weekly, with provision for dose escalation or reduction according to the toxicity observed. Of 29 evaluable patients, one patient achieved a complete response of 19+ weeks duration, and ten patients achieved a partial response with a median duration of 22 weeks (range 16-48), for a total response rate of 38%. Piritrexim was generally well tolerated, with myelosuppression as the major toxicity, that frequently required dose modification. We conclude that piritrexim appears to be an active agent in patients with metastatic urothelial cancer when administered as a 5-day, low-dose oral schedule. It would be attractive to investigate the combination of piritrexim and cisplatin.


Br. J. Cancer (1993), 67, 388–390 © Macmillan Press Ltd., 1993

### Oral piritrexim, an effective treatment for metastatic urothelial cancer

R. de Wit[1], S.B. Kaye[2], J.T. Roberts[3], G. Stoter[1], J. Scott[4] & J. Verweij[1]

[1]*Department of Medical Oncology, Rotterdam Cancer Institute (Dr. Daniel den Hoed Kliniek), PO Box 5201, 3008 AE Rotterdam, The Netherlands;* [2]*Beatson Oncology Centre, Western Infirmary, Glasgow G11 6NT;* [3]*Newcastle General Hospital, Newcastle-upon-tyne, NE4 6BE;* [4]*Wellcome Research Laboratories, Langley Court, South Eden Park Road, Beckenham, Kent BR3 3BS, UK.*

**Summary** Piritrexim is a lipid-soluble inhibitor of dihydrofolate reductase (DHFR) that enters tumour cells rapidly by passive diffusion, cannot be polyglutamated, and is as effective as methotrexate in inhibiting DHFR. Bioavailability after oral dosing is approximately 75%. We performed a phase II study with oral piritrexim in non-chemotherapy pretreated patients with metastatic urothelial cancer.

Thirty-three patients were treated with 25 mg three times daily for 5 consecutive days, repeated weekly, with provision for dose escalation or reduction according to the toxicity observed.

Of 29 evaluable patients, one patient achieved a complete response of 19+ weeks duration, and ten patients achieved a partial response with a median duration of 22 weeks (range 16–48), for a total response rate of 38%. Piritrexim was generally well tolerated, with myelosuppression as the major toxicity, that frequently required dose modification.

We conclude that piritrexim appears to be an active agent in patients with metastatic urothelial cancer when administered as a 5-day, low-dose oral schedule. It would be attractive to investigate the combination of piritrexim and cisplatin.

Piritrexim (2,4 - diamino - 6[2,5 - dimethoxybenzyl] - 5 - methyl pyrido - [2,3d] pyrimidine; PTX) is a new, lipid-soluble dihydrofolate reductase (DHFR) inhibitor (Duch *et al.*, 1982; Sedwick *et al.*, 1982; Sigel *et al.*, 1987). The drug differs from methotrexate (MTX) in that it crosses membranes rapidly by passive diffision and cannot be polyglutamated, therefore the intracellular levels are expected to parallel plasma levels in highly perfused tissues. PTX is as potent as MTX *in vitro* as an inhibitor of DHFR. Bioavailability after oral dosing is approximately 75% (Weiss *et al.*, 1989). The relatively short half-life (3 to 5 h) and the lack of polyglutamation of PTX makes a continuous administration of this agent attractive. Consequently, phase I and II studies of PTX were conducted using prolonged, low-dose oral schedules (Feun *et al.*, 1991*a,b*). Myelosuppression proved to be dose-limiting. Antitumour activity was observed in patients with malignant melanoma and urothelial cancer (Weiss *et al.*, 1989; Feun *et al.*, 1991*a,b*). The recommended dose for further phase II studies was 25 mg three times a day for 5 days (Feun *et al.*, 1991*b*). We performed a phase II study with oral PTX in non-chemotherapy pretreated patients with metastatic urothelial cancer.

### Patients and methods

### *Patients*

Eligibility criteria required histologically proven transitional cell carcinoma of the urinary tract, measurable distant metastases or measurable pelvic tumour not amenable to locoregional treatment, performance status (WHO Scale) 0–2, serum creatinine below 140 $\mu$mol $l^{-1}$, bilirubin below 25 $\mu$mol $l^{-1}$, white blood cells (WBC) above $4 \times 10^9 l^{-1}$ and platelets above $100 \times 10^9 l^{-1}$. Patients with prior systemic chemotherapy, irradiated indicator lesions or brain metastases, or with poor medical risk were excluded.

### *Study design*

PTX was administered orally at a dose of 25 mg three times daily for 5 days, repeated weekly. The drug was administered 1 h before meals or 2 h after meals. There was a provision for dose escalation to 25 mg four times daily for 5 days if no toxicity after a set of four cycles had been encountered. The dose was unchanged if grade 1 myelotoxicity had been experienced in a set of four cycles. There was a dose delay in case of WHO grade 2 myelotoxicity within the first 3 weeks, and grade 3/4 at any time. After recovery, treatment was resumed with 25 mg three times daily for 4 days (one step), with provision for a second dose reduction to 25 mg twice daily for 4 days (second step). Patients were seen weekly at the outpatient clinic in order to document adverse events and to make dose adjustments. Measurement of the indicator lesion(s) was performed every 4 weeks. Response was assessed according to WHO criteria; a complete response (CR) was defined as the complete disappearance of all known disease, determined by two observations not less than 4 weeks apart; partial response (PR) as at least 50% reduction in the sum of the products of the two largest perpendicular diameters of all measurable lesions, determined by two observations not less than 4 weeks apart; progressive disease (PD) as an increase of at least 25% in any measurable lesion or the appearance of a new lesion; and no change (NC) as less than 50% reduction in total tumour volume or less than 25% increase in any measurable lesion. Response duration was calculated from the start of chemotherapy to the date of first observation of progressive disease.

In case of response (CR/PR), patients continued treatment until progression. In case of PD at 4 or 8 weeks, patients went off study, and were offered the option of cisplatin combination chemotherapy. Patients were evaluable for response if they had completed 8 weeks of treatment, unless there was progression at 4 weeks. All patients who had received at least one dose of chemotherapy were evaluable for toxicity, which was also graded according to WHO criteria.

Institutional review board-approved informed consent was obtained for all patients before study entry.

### Results

Thirty-three patients were entered into the study. Patients characteristics are shown in Table I. Four patients were not evaluable for response: one patient was diagnosed to have a second primary (lung) during the course of treatment, and three patients stopped before 4 weeks due to toxicity (one patient had grade 4 leucopenia after 3 weeks and two

Correspondence: R. de Wit, Department of Medical Oncology, Rotterdam Cancer Institute, P.O. Box 5201, 3008 AE Rotterdam, The Netherlands.
Received 27 August 1992; and in revised form 30 September 1992.

**Table I** Patient characteristics

| | | |
|---|---|---|
| Number of patients entered | | 33 |
| Sex (male/female) | | 22/11 |
| Age median (range) | | 65 (41–76) |
| WHO Performance Score 0/1/2 | | 10/19/4 |
| Prior treatment | surgery | 10 |
| | radiotherapy | 17 |
| | none | 6 |
| Sites of disease: | primary tumour (including local recurrence) | 8 |
| | lymph nodes | 20 |
| | lung | 9 |
| | liver | 6 |
| | bone | 5 |
| | skin | 1 |

patients developed grade 2/3 skin toxicity after 2 and 3 weeks respectively). Therefore 29 patients were evaluable for response. All 33 patients were considered evaluable for toxicity. The median duration of treatment was 8 weeks (range 3–47).

Of the 29 evaluation patients, one patient with extensive lymph node metastases achieved a complete response, of 19+ weeks duration, and ten patients achieved a partial response, with a median response duration of 22 weeks (range 16–48), for a total response rate of 38% (95% confidence interval 20–56). Sites of response were; lung 5, liver 1, lymph nodes 6 and primary 1. Ten patients had NC at 8 weeks, of whom seven were kept on PTX for a median of 15 weeks (range 9–18), and seven patients had progressive disease.

Thirteen patients were crossed over to cisplatin based chemotherapy, cisplatin/methotrexate (DDP/MTX) (Yagoda, 1987); of these one achieved a CR, four a PR, three had NC, and four had PD. One patient was not evaluable. Of these five responders on DDP/MTX, one had initially also responded to PTX, one had NC at 8 weeks, and two had progressed during PTX treatment. The fifth patient was not evaluable to response due to grade 4 leucopenia at 3 weeks and subsequent cessation of PTX treatment.

### *Toxicity*

The most frequent toxicities are listed in Table II. The major toxicities were leukopenia and thrombocytopenia, which however were manageable and rapidly reversible; recovery from nadir was usually reached within a few days after treatment interruption and never required more than 7 days. There were no episodes of bleeding or leucopenic fever. In six patients grade 3–4 myelotoxicity developed quite suddenly within 1 treatment week, after preceding grade 0–1 toxicity, whereas spontaneous recovery from grade 1 toxicity without simultaneous dose adjustment was also observed. This necessitated continuation of weekly determination of blood counts and appropriate dose adjustments. Three patients developed a maculo-papular rash within the first weeks of treatment, that required cessation of PTX treatment in two cases. These skin rashes resolved completely within days after PTX was stopped. No mucositis was observed. Mild nausea and vomiting was seen occasionally in 52% of the patients, and only a minority of patients used anti-emetic drugs at regular intervals. There was one case of dyspnea and evidence of interstitial pulmonary changes, which were reversible upon cessation of treatment and was attributed to PTX. The clinical details of this patient are in press elsewhere (De Wit *et al.*, 1992). In three patients we noted the development of low grade hemolytic anaemia during the course of treatment that was rapidly reversible upon cessation of PTX. An example is shown in Figure 1. To our knowledge this side effect has not been reported previously. Dose escalation was performed only in three patients. In view of the myelotoxicity observed in the first five patients, no further dose escalations were performed. A one step dose reduction was performed in 13 patients, and a second step was made in seven patients.

**Table II** Toxicity

| | *Grade (WHO)* | | | | |
|---|---|---|---|---|---|
| *Worst toxicity observed* | *0* | *1* | *2* | *3* | *4* |
| Leucocytes | 9 | 7 | 13 | 3 | 1 |
| Platelets | 17 | 1 | 6 | 6 | 3 |
| Nausea/vomiting | 16 | 9 | 8 | 0 | 0 |
| Skin | 30 | 1 | 1 | 1 | 0 |
| Pulmonary | 32 | 0 | 1 | 0 | 0 |
| Renal | 31 | 2 | 0 | 0 | 0 |
| Liver | 32 | 0 | 1 | 0 | 0 |

### Discussion

Presently, DDP and MTX are the most commonly used single agents in the treatment of advanced transitional cell cancer of the urothelial tract, with average response rates of 24% in 629 patients and 29% in 236 patients, respectively. The responses were usually partial and of short duration (median 3 to 6 months) (Yagoda, 1987; Oliver *et al.*, 1986; Khandekar *et al.*, 1985; Troner *et al.*, 1987; Hillcoat *et al.*, 1989; Loehrer *et al.*, 1990). In an attempt to improve the results various combinations of DDP and MTX with or

**Figure 1** An example of hemolysis during PTX treatment, black arrows indicate blood transfusions (units of packed cells).

without other agents have been studied. Although with combination chemotherapy response rates have increased to 40–70%, the median duration of response and survival is still less than 1 year, whereas such intensive combination chemotherapy is at the cost of considerable toxicity (Oliver et al., 1986; Khandekar et al., 1985; Troner et al., 1987; Hillcoat et al., 1989; Loehrer et al., 1990; Stoter et al., 1987; Harker et al., 1985; Yagoda, 1989; Sternberg et al., 1989; Logothetis et al., 1990a, b). Apart from the limited efficacy of the currently available agents, intercurrent disease in this usually elderly population may preclude treatment with drugs such as cisplatin and methotrexate due to renal insufficiency, or doxorubicin due to cardiac disease. These factors warrant the search for new effective agents (even in first-line treatment). This phase II study investigated the clinical usefulness of PTX, a new lipid-soluble dihydrofolate reductase inhibitor, in patients with advanced urothelial cancer. Our results reported here indicate that PTX administered in a 5-day, low-dose oral schedule is active in urothelial cancer. Of 29 evaluable patients treated with PTX, one achieved a CR and 10 a PR, with a median response duration of 22 weeks, for a total response rate of 38% (95% confidence interval 20–56). When the non-evaluable patients are included in the analysis, the overall response rate is 33%. This response rate is similar to that for the parent compound methotrexate: 29% (95% confidence interval 23–35%).

PTX was generally well tolerated. The major toxicity was myelosuppression. Other toxicities included skin rash that required early cessation of PTX treatment in two cases, and mild nausea and vomiting. Three patients developed hemolytic anaemia during treatment, and one patient developed reversible interstitial pulmonary changes, that were attributed to PTX. In several patients we observed the sudden development of myelotoxicity, after prolonged treatment periods without significant previous myelotoxicity, whereas other patients demonstrated spontaneous recovery from grade 1 toxicity despite treatment continuation. This may be indicative of intra-patient variability of drug resorption, and because of its unpredictable nature necessitated weekly determinations of blood counts (Weiss et al., 1989). Without exceptions all side-effects were rapidly reversible after discontinuation of drug administration.

In conclusion, PTX appears to be an active agent in patients with advanced urothelial cancer when administered as a 5-day, low-dose oral schedule. Furthermore, the drug is well tolerated with myelosuppression as the major toxicity, that is sometimes unpredictable and frequently requires dose modification. It would be attractive to investigate the combination of PTX and cisplatin.

This research was supported in part by a grant from Wellcome, Beckenham, UK.

The authors wish to thank Miss Thérèse van Eijk for typing the manuscript.

### References

DE WIT, R., VERWEIJ, J., SLINGERLAND, R. & STOTER, G. (1992). Piritrexim induced pulmonary toxicity. *Am. J. Clin. Oncol.* (in press).

DUCH, D.S., EDELSTEIN, M.P., BOWERS, S.W. & NICHOLS, C.A. (1982). Biochemical and chemotherapeutic studies in 2,4-diamino-6 (2,5-dimethoxybenzyl)-5-methyl pyrido (2,3d) pyrimidine (BW 30lu). A novel lipid-soluble inhibitor of dihydrofolate reductase. *Cancer Res.*, **42**, 3987–3994.

FEUN, L.G., SAVARAJ, N., BENEDETTO, P., HANLON, J., SRIDHAR, K.S., COLLIER, M., RICHMAN, S., LIAO, S.H. & CLENDENINN, N.J. (1991a). Phase I trial of piritrexim capsules using prolonged, low-dose oral administration for the treatment of advanced malignancies. *J. Natl Cancer Inst.*, **83**, 51–55.

FEUN, L.G., GONZALEZ, R., SAVARAJ, N., HANLON, J., COLLIER, M., ROBINSON, W.A. & CLENDENINN, N.J. (1991b). Phase II trial of piritrexim in metastatic melanoma using intermittent, low-dose administration. *J. Clin. Oncol.*, **9**, 464–467.

HARKER, W.G., MEYERS, F.J., FREIHA, F.S., PALMER, J.M., SHORTLIFFE, L.D., HANNIGAN, J.F., MCWHIRTER, K.M. & TORTI, F.M. (1985). Cisplatin, methotrexate and vinblastine (CMV): and effective chemotherapy regimen for metastatic transitional cell carcinoma of the urinary tract. A Northern California Oncology Group study. *J. Clin. Oncol.*, **3**, 1463–1470.

HILLCOAT, B.L., RAGHAVAN, D., MATTHEWS, J., KEFFORD, R., YUEN, K., WOODS, R., OLVER, I., BISHOP, J., PEARSON, B., COOREY, G., LEVI, J., ABBOTT, R.L., ARONEY, R., GILL, P.G. & MCLENNAN, R. (1989). A randomized trial of cisplatin versus cisplatin plus methotrexate in advanced cancer of the urothelial tract. *J. Clin. Oncol.*, **7**, 706–709.

KHANDEKAR, J.D., ELSON, P.V., DEWYS, W.D., SLAYTON, R.E. & HARRIS, D.T. (1985). Comparative activity and toxicity of cis-diamminedichloroplatinum (DDP) and a combination of doxorubicin, cyclophosphamide, and DDP in disseminated transitional cell carcinomas of the urinary tract. *J. Clin. Oncol.*, **3**, 539–545.

LOEHRER, P.J., ELSON, P., KUEBLER, J.P., CRAWFORD, E.D., TANNOCK, I., RAGHAVAN, D., STUART-HARRIS, R., TRUMP, D. & EINHORN, L.H. (1990). Advanced bladder cancer: a prospective intergroup trial comparing single agent cisplatin (CDDP) versus M-VAC combination therapy. *Proc. ASCO*, **9**, 132.

LOGOTHETIS, C.J., DEXEUS, F.H., SELLA, A., AMATO, R.J., KILBOURN, R.G., FINN, L. & GUTTERMAN, J.U. (1990a). Escalated therapy for refractory urothelial tumours: methotrexate-vinblastine-doxorubicin-cisplatin plus unglycosylated recombinant human granulocyte-macrophage colony-stimulating factor. *J. Natl Cancer Inst.*, **82**, 667–672.

LOGOTHETIS, C.J., DEXEUS, F.H., FINN, L., SELLA, A., AMATO, R.J., AYALA, A.G. & KILBOURN, R.G. (1990b). A prospective randomized trial comparing M-VAC and CISCA chemotherapy for patients with metastatic urothelial tumours. *J. Clin. Oncol.*, **8**, 1050–1055.

OLIVER, R.T.D., KWOK, H.K., HIGHMAN, W.J. & WAXMAN, J. (1986). Methotrexate, cisplatin and carboplatin as single agents and in combination for metastatic bladder cancer. *Br. J. Urol.*, **58**, 31–35.

SEDWICK, W.D., HAMRELL, M., BROWN, O.E. & LAZLO, J. (1982). Metabolic inhibition by a new antifolate 2,4-diamino-6 (2,5-dimethoxybenzyl)-5-methyl pyrido (2,3d) pyrimidine (BW30lu), an effective inhibitor of human lymphoid and dehydrofolate reductase-over producing mouse cell lines. *Mol. Pharmacol.*, **22**, 766–770.

SIGEL, C.W., MACKLIN, A.W., WOOLLEY, J.L., JOHNSON, N.W., COLLIER, M.A., BLUM, M.R., CLENDENINN, N.J., EVERITT, B.J.M., GREBE, G., MACKARS, A., FOSS, R.G., DUCH, D.S., BOWERS, S.W. & NICHOL, C.A. (1987). Preclinical biochemical pharmacology and toxicology of piritrexim, a lipophile inhibitor of dehydrofolate reductase. *NCI Monogr.*, **5**, 111–120.

STERNBERG, C.N., YAGODA, A., SCHER, H.I., WATSON, R.C., HERR, H.W., MORSE, M.J., SOGANI, P.C., VAUGHAN, E.D., BANDER, N., WEISELBERG, L.R., GELLER, N., HOLLANDER, P.S., LIPPERMAN, R., FAIR, W.R. & WHITMORE, W.F. (1989). Methotrexate, vinblastine, doxorubicin and cisplatin for advanced transitional cell carcinoma of the urothelium. *Cancer*, **64**, 2448–2458.

STOTER, G., SPLINTER, T.A.W., CHILD, J.A., FOSSA, S.D., DENIS, L., VAN OOSTEROM, A.T., DE PAUW, M. & SYLVESTER, R. (1987). Combination chemotherapy with cisplatin and methotrexate in advanced transitional cell cancer of the bladder. *J. Urol.*, **137**, 663–667.

TRONER, M., BIRCH, R., OMURA, G.A. & WILLIAMS, S. (1987). Phase III comparison of cisplatin alone versus cisplatin, doxorubicin and cyclophosphamide in the treatment of bladder (urothelial) cancer: a Southeaster Cancer Study Group Trial. *J. Urol.*, **137**, 660–662.

WEISS, G.R., SAROSY, A.G., SHENKENBERG, T.D., WILLIAMS, T., CLENDENINN, N.J., VON HOFF, D.D., WOOLLEY, J.L., LIAO, S.H.T. & BLUM, M.R. (1989). A phase I clinical and pharmacological study of weekly intravenous infusions of piritrexim (BW301U). *Eur. J. Cancer Clin. Oncol.*, **25**, 1867–1873.

YAGODA, A. (1987). Chemotherapy of urothelial tract tumours. *Cancer*, **60**, 574–585.

YAGODA, A. (1989). The role of cisplatin-based chemotherapy in advanced urothelial tract cancer. *Sem. Oncol.*, **16** (Suppl. 6) 98–104.